# Supplementary material for: The human RIF1-Long isoform interacts with BRCA1 to promote recombinational fork repair under DNA replication stress
Source: Nat Commun. 2025 Jul 1;16:5820. doi: 10.1038/s41467-025-60817-y (PMC12214830; doi:10.1038/s41467-025-60817-y)
Supplement: Supplementary file 1 — Supplementary Information [file 41467_2025_60817_MOESM1_ESM.pdf]

The human RIF1-Long isoform interacts with BRCA1 to promote recombinational fork repair under DNA replication stress

Supplementary information

Supplementary Figures 1 – 7

Supplementary Table 1

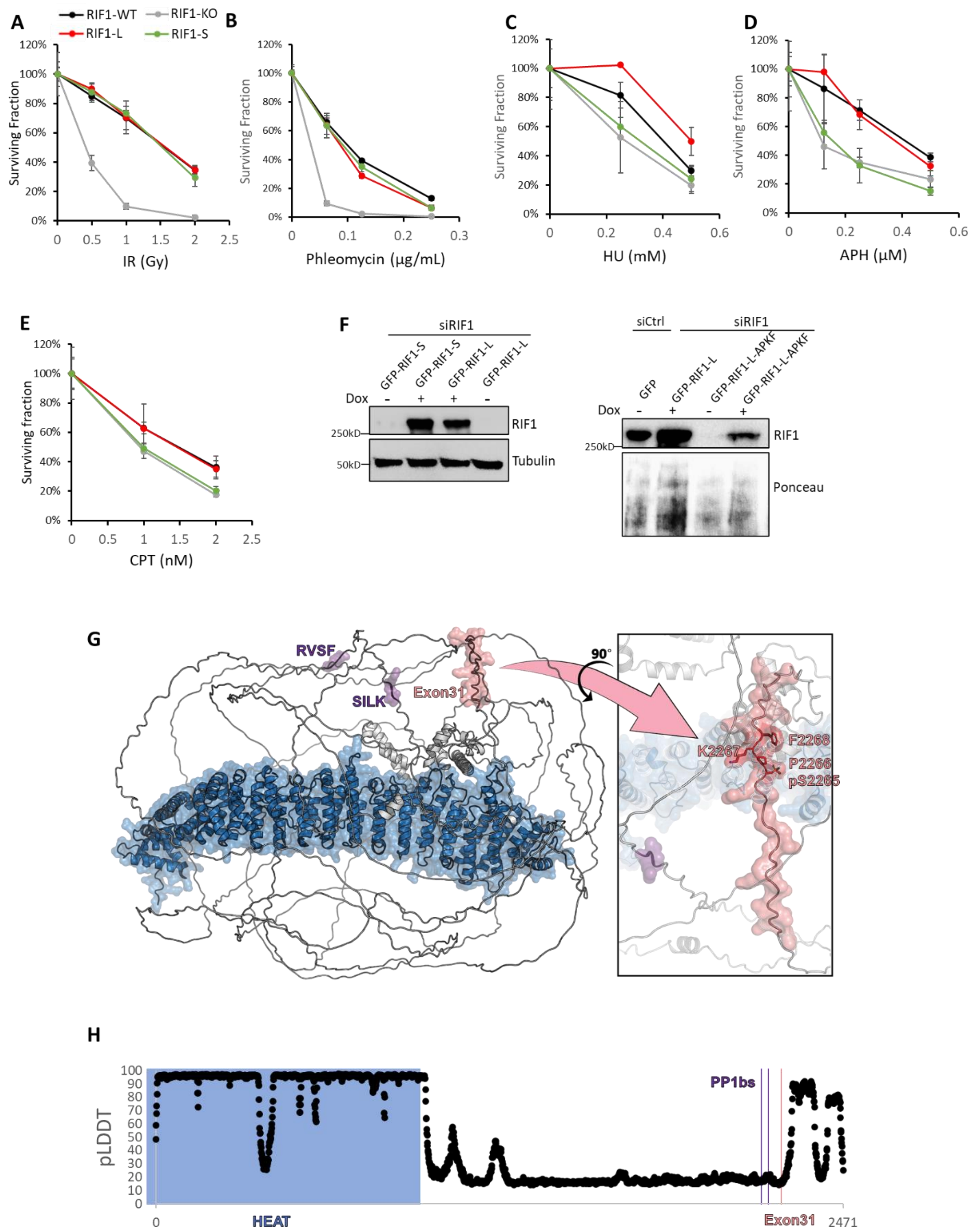

Supplementary Figure 1. Cells expressing only RIF1-L or only RIF1-S exhibit differential sensitivity to replication stress reagents

(A)-(E) Clonogenic survival assay of HCT116 cells with indicated treatments (cell lines described previosuly[24]). IR – ionising radiation; HU – hydroxyurea; APH – aphidicolin; CPT – camptothecin. Percentage survival values are normalised to the no drug treatment control. Means and standard errors of technical triplicates (n=3) are plotted. Source data are provided as a Source Data file.

(F) Western blot analysis of ectopic GFP-RIF1 expression in Flp-In TREx 293 cells (cell lines described previosuly[24]). siRIF1 was used to deplete endogenous RIF1. The ectopic GFP-RIF1 transcripts are resistant to siRIF1, due to specific mutations in the GFP-RIF1 construct. Doxycycline was used to induce expression of the GFP-RIF1 constructs.

(G) Cartoon representation of an Alphafold2 predicted model of the RIF1-L protein coloured as for the schematic representation with surfaces displayed for the HEAT repeats (blue), PP1 interacting motifs (purple) and exon 31 (pink). The inset shows the positioning of the SPKF motif within exon-31.

(H) pLDDT (predicted local distance difference) plot for presented Alphafold2 model, coloured as before, demonstrating the region corresponding to exon 31 is predicted to be disordered and therefore readily accessible for phosphorylation and subsequent interactions.

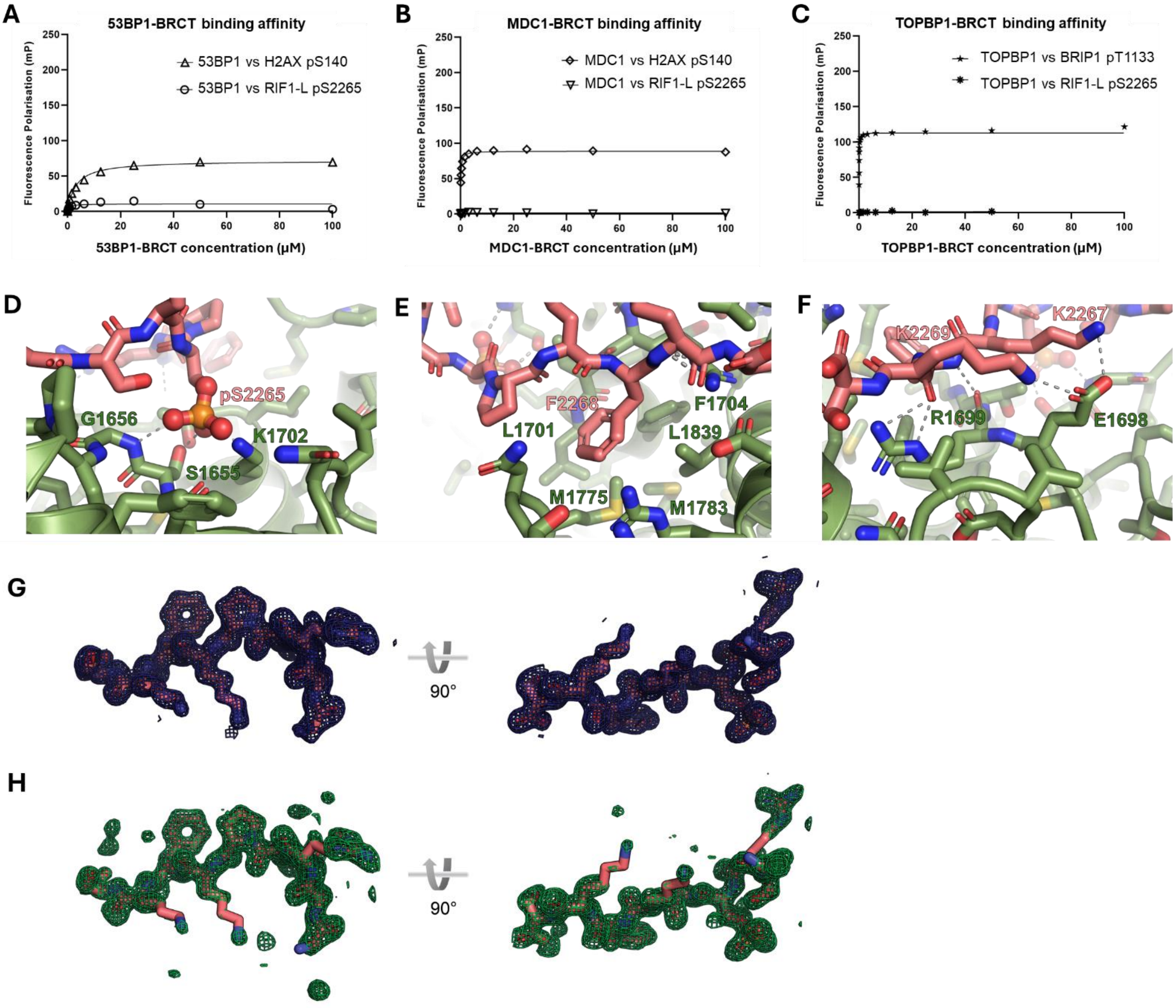

Supplementary Figure 2. RIF1-L phospho-SPKF peptide binds to tandem BRCT domain of BRCA1 *in vitro*

(A) Fluorescence polarisation analysis testing binding of the tandem BRCT domains of 53BP1 to RIF1-L phosphoS^{2265}PKF peptide. Binding to an H2AX pS140 peptide (an established phospho-recognition site for 53BP1 BRCT) is shown for comparison.
(B) Fluorescence polarisation analysis testing binding of the tandem BRCT domains of MDC1 to RIF1-L phosphoS^{2265}PKF peptide. Binding to an H2AX pS140 peptide (an established phospho-recognition site for MDC1 BRCT) is shown for comparison.
(C) Fluorescence polarisation analysis testing binding of the tandem BRCT domains of TOPBP1 to RIF1-L phosphoS^{2265}PKF peptide. Binding to an BRIP1 pT1133 peptide (an established phospho-recognition site for TOPBP1 BRCT) is shown for comparison.
(D) The phosphate group of RIF1-L-pS2265 is found in the canonical phosphate binding pocket of the BRCA1 BRCTs and recognised by a network of hydrogen bonding and ionic interactions centred around the side chains of S1655, K1702, and the main chain of G1656.
(E) Common to other structures of BRCA1 bound to phosphorylated peptides, RIF1 F2268, in the +3 position of the peptide, is found providing a second anchor point in the deep hydrophobic pocket formed in the BRCA1 BRCT module by residues L1701, F1704, M1775, M1783 and L1839.
(F) Additional interactions are formed between the RIF1-L peptide and the BRCA1 BRCT domains, including between E1698 of BRCA1 bonding with K2267 and K2269 of RIF1-L and the mainchain of F2268 in the BRCT domains with the main chain and side chain of R1699 in the peptide.
(G) **(H)** Orthogonal views of the region of the RIF1-L peptide modelled in the structure (pink sticks) displayed alongside corresponding electron density (G) the 2Fo-Fc map contoured at 1.5σ is shown as blue mesh surface (H) the Fo-Fc omit map calculated using PHENIX Polder with annealing contoured at 3σ shown as green mesh surface.

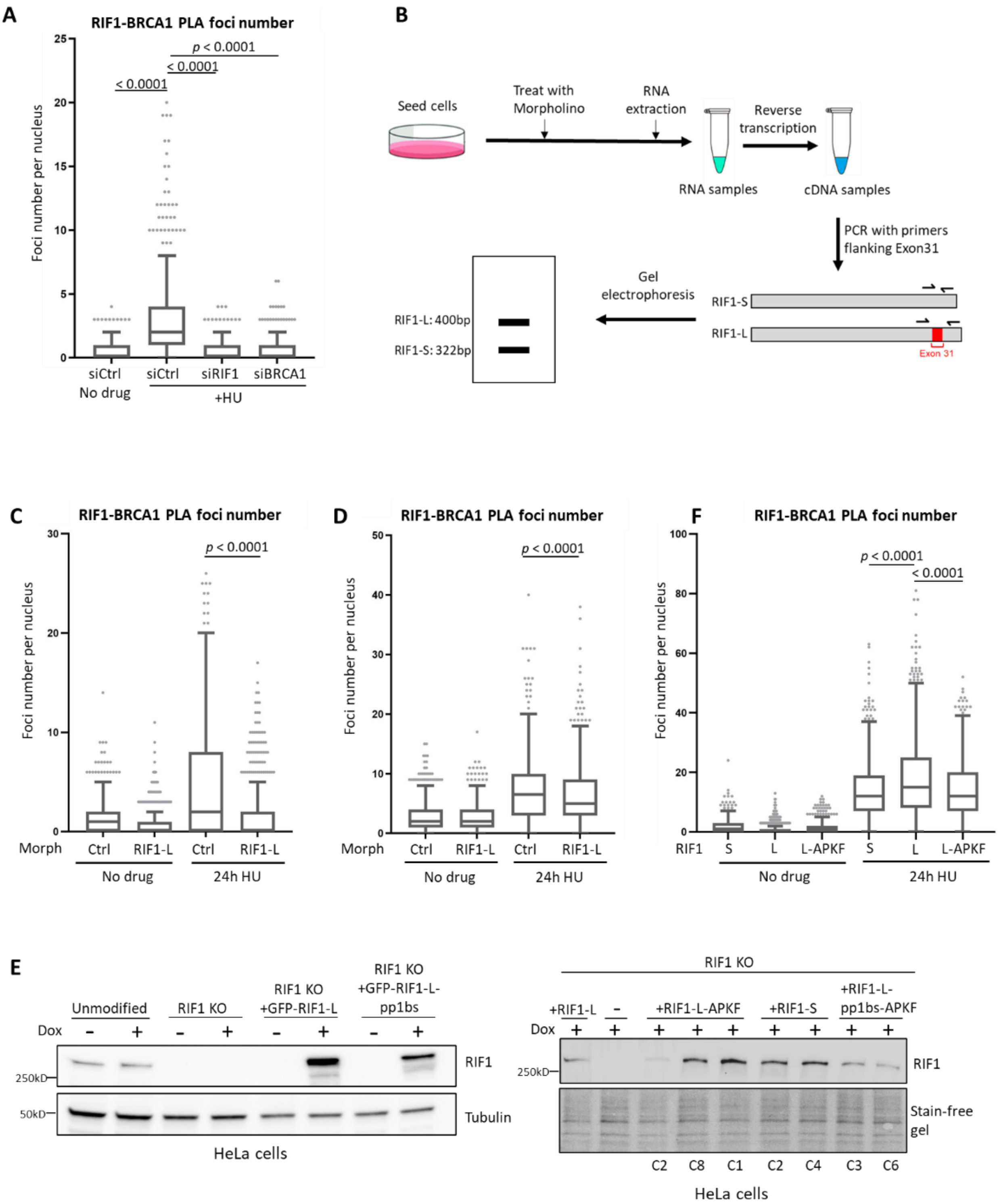

Supplementary Figure 3. RIF1-L isoform is required for proximity with BRCA1 *in vivo*

(A) RIF1-BRCA1 PLA analysis in RPE-1 cells with indicated treatments. HU: 4mM 24hr. *p* values calculated by Kruskal-Wallis test with Dunn's multiple comparisons using the 'siCtrl +HU' sample as the control group.
(B) Experimental procedure for the gel analysis shown in Fig.3E. RPE-1 cells were treated with Control or RIF1-L Morpholinos for 48hr. RNA was extracted and reverse transcribed to cDNA, which was used to template PCR reactions with a pair of primers flanking Exon 31. PCR products were analysed in gel electrophoresis: the upper band corresponds to RIF1-L transcript while the lower band corresponds to RIF1-S transcript.
(C) RIF1-BRCA1 PLA analysis in RPE-1 cells with indicated Morpholino and drug treatments. Morph: abbreviation for Morpholino. HU: 4mM 24hr. (Repeat of Fig.3G). *p* values (two-tailed) calculated by Mann-Whitney test.
(D) RIF1-BRCA1 PLA analysis in U2OS cells with indicated Morpholino and drug treatments. HU: 4mM 24hr.
(E) Western blot analysis of RIF1 expression in HeLa cells (cell lines previously described[41]). HeLa cells were either RIF1 KO, or RIF1 KO supplemented with Dox-inducible GFP-tagged RIF1 derivatives. In the right panel, 'C' indicates clone ID. C8 of '+RIF1-L-APKF', C2 of '+RIF1-S', C3 of '+RIF1-L-pp1bs-APKF' were selected for experiments presented in this study.
(F) RIF1-BRCA1 PLA analysis in HeLa cells that express only RIF1-S, or RIF1-L, or RIF1-L-APKF, as described in (E). HU: 4mM 24hr. *p* values calculated by Kruskal-Wallis test with Dunn's multiple comparisons between indicated groups.
*In the Tukey box-and-whisker plots in this and all following figures, n numbers of samples are listed in Supplementary Data 1. Numbers of independent experimental repeats is stated in 'Statistics and Reproducibility' section. Source data are provided as a Source Data file.

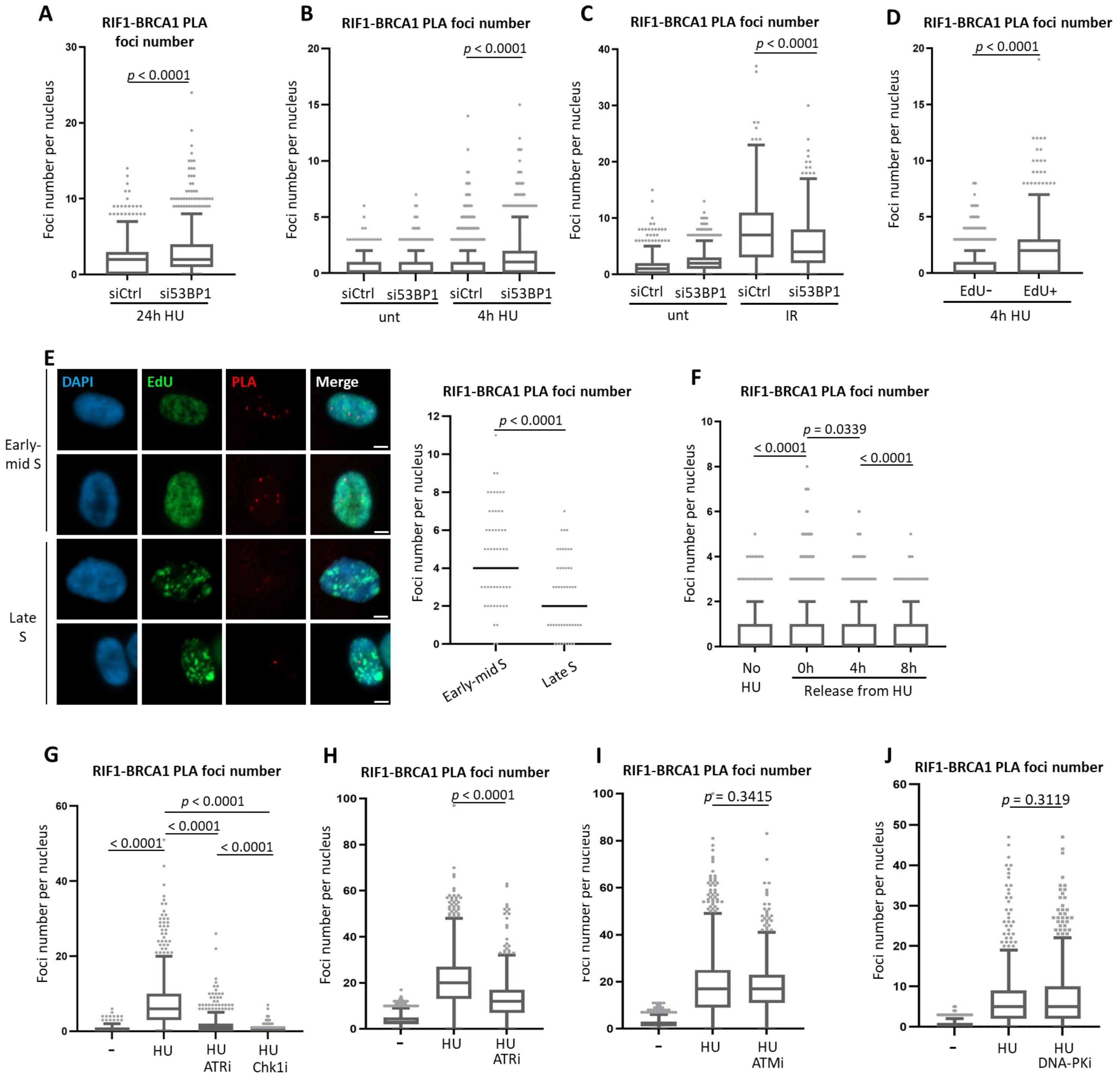

Supplementary Figure 4. RIF1-BRCA1 proximity occurs in S phase and depends on ATR signaling

(A) (B) (C) RIF1-BRCA1 PLA analysis in Control or 53BP1-depleted RPE-1 cells with indicated treatments. HU: 4mM for 24hr in (A) and for 4hr in (B). IR in (C): 10Gy. *p* values (two-tailed) calculated by Mann-Whitney test.

(D) RIF1-BRCA1 PLA foci analysis in RPE-1 cells, following the experiment scheme described in Fig.4C. (Repeat of Fig.4D). *p* values (two-tailed) calculated by Mann-Whitney test.

(E) Representative images of EdU-positive RPE-1 cells in the RIF1-BRCA1 PLA experiment described in Fig.4C. Cells were classified as in 'early-mid S' or 'late S' phase based on the EdU pattern[42]. Scalebar: 5μm. RIF1-BRCA1 PLA foci number was quantified. *p* values (two-tailed) calculated by Mann-Whitney test.

(F) RIF1-BRCA1 PLA analysis in RPE-1 cells collected at indicated timepoints after removal of HU, following the experiment scheme described in Fig.4E (Repeat of Fig.4E). *p* values calculated by Kruskal-Wallis test with Dunn's multiple comparisons between indicated groups.

(G) RIF1-BRCA1 PLA analysis in RPE-1 cells with indicated kinase inhibitor and HU treatments. HU: 4mM 24hr; ATRi (VE-821): 1μM 24hr; Chk1i (PF-477736): 1μM 24hr. (Repeat of Fig.3F). *p* values calculated by Kruskal-Wallis test with Dunn's multiple comparisons between indicated groups.

(H) (I) RIF1-BRCA1 PLA analysis in HeLa +RIF1-L cells with indicated kinase inhibitor and HU treatments. HU: 4mM 24hr; ATRi (VE-821): 1μM 24hr; ATMi (KU-60019): 2μM 24hr. (HeLa cells described in Fig.S3E). *p* values (two-tailed) calculated by Mann-Whitney test.

(J) RIF1-BRCA1 PLA analysis in RPE-1 cells with indicated kinase inhibitor and HU treatments. HU: 4mM 24hr; DNA-PKi (NU-7441): 1μM 24hr. Experiment was repeated twice, and one representative result is shown. *p* values (two-tailed) calculated by Mann-Whitney test.

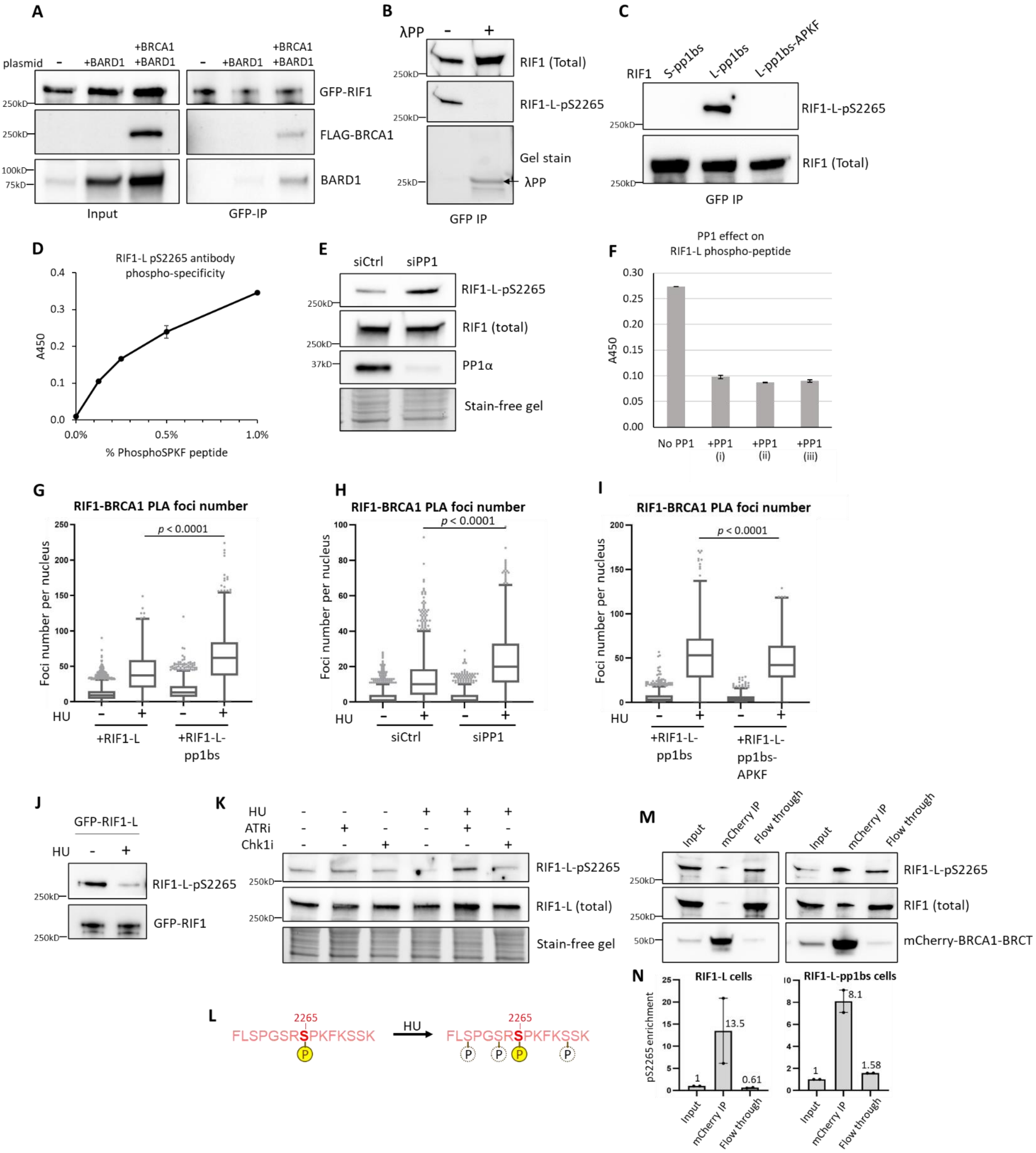
A
plasmid
-
+BARD1
+BRCA1 +BARD1
GFP-RIF1
FLAG-BRCA1
BARD1
250kD
100kD
75kD
Input
GFP-IP
B
λPP
-
+
RIF1 (Total)
RIF1-L-pS2265
Gel stain
λPP
25kD
GFP IP
C
RIF1
S-pp1bs
L-pp1bs
L-pp1bs-APKF
RIF1-L-pS2265
RIF1 (Total)
GFP IP
D
RIF1-L pS2265 antibody phospho-specificity
A450
% PhosphoSPKF peptide
0.0%
0.5%
1.0%
E
siCtrl
siPP1
RIF1-L-pS2265
RIF1 (total)
PP1α
37kD
Stain-free gel
F
PP1 effect on RIF1-L phospho-peptide
A450
No PP1
+PP1 (i)
+PP1 (ii)
+PP1 (iii)
G
RIF1-BRCA1 PLA foci number
Foci number per nucleus
p < 0.0001
HU
+RIF1-L
+RIF1-L-pp1bs
H
RIF1-BRCA1 PLA foci number
Foci number per nucleus
p < 0.0001
HU
siCtrl
siPP1
I
RIF1-BRCA1 PLA foci number
Foci number per nucleus
p < 0.0001
HU
+RIF1-L-pp1bs
+RIF1-L-pp1bs-APKF
J
GFP-RIF1-L
HU
RIF1-L-pS2265
GFP-RIF1
K
HU
ATRi
Chk1i
RIF1-L-pS2265
RIF1-L (total)
Stain-free gel
L
2265
FLSPGSRSPKFKSSK
HU
2265
FLSPGSRSPKFKSSK
M
Input
mCherry IP
Flow through
RIF1-L-pS2265
RIF1 (total)
mCherry-BRCA1-BRCT
50kD
N
RIF1-L cells
RIF1-L-pp1bs cells
pS2265 enrichment
1
13.5
0.61
1
8.1
1.58
Input
mCherry IP
Flow through

Supplementary Figure 5. RIF1-L-BRCA1 interaction is suppressed by PP1-mediated RIF1-S2265 dephosphorylation

(A) RIF1-BRCA1-BARD1 co-IP analysis. Flp-In TREx 293 cells expressing GFP-RIF1-L-pp1bs were transfected with a FLAG-BRCA1 plasmid and a BARD1 plasmid. GFP IP was performed and immunoblotted for GFP-RIF1, FLAG-BRCA1, and BARD1.

(B) Validation of the RIF1-L-pS2265 antibody by western blot analysis. GFP-immunoprecipitated fraction was acquired from Flp-In TREx 293 cells expressing GFP-RIF1-L-pp1bs, and treated without or with λ Protein Phosphatase (λPP). Immunoblotting with the RIF1-L-pS2265 antibody was performed on the GFP-IP samples.

(C) Validation of the RIF1-L-pS2265 antibody by western blot analysis. GFP-immunoprecipitated fraction was acquired from Flp-In TREx 293 cells expressing GFP-RIF1-S-pp1bs, GFP-RIF1-L-pp1bs, or GFP-RIF1-L-pp1bs-APKF. Immunoblotting with the RIF1-L-pS2265 antibody was performed on the GFP-IP samples.

(D) Validation of the RIF1-L-pS2265 antibody by ELISA assay. Two biotinylated peptides corresponding to RIF1-L exon 31, with or without phosphorylation at S^{2265} were mixed in different ratios (*x* axis shows the percentage of phosphoSPKF peptide in the mixture). They were captured in streptavidin-coated wells and incubated with the RIF1-L-pS2265 antibody. The amount of bound pS2265 antibody was measured using HRP-conjugated secondary antibody.

(E) Western blot analysis of RIF1-L S2265 phosphorylation in HeLa cells treated with siControl or siPP1. siPP1 was a combination of siPP1α, siPP1β, and siPP1γ; used at a total concentration of 50nM.

(F) ELISA analysis (as described in (D)) of PP1-mediated dephosphorylation of RIF1-L S^{2265}. RIF1-L phosphoSPKF peptide was incubated without or with purified human PP1 protein. Three independent dilutions of PP1 (i) (ii) (iii) were tested. Phosphorylation was quantified using the ELISA assay. Mean and standard errors of technical triplicates were plotted.

(G) RIF1-BRCA1 PLA foci analysis in HeLa cells with indicated RIF1 expression and treatments. (HeLa cells described in Fig.S3E). HU: 4mM 24hr. (Repeat of Fig.5G). *p* values (two-tailed) calculated by Mann-Whitney test.

(H) RIF1-BRCA1 PLA foci analysis in HeLa cells treated with siControl or siPP1. siPP1 was a combination of siPP1α, siPP1β, and siPP1γ; used at a total concentration of 50nM. HU: 4mM 24hr. *p* values calculated by (two-tailed) Mann-Whitney test.

(I) RIF1-BRCA1 PLA foci analysis in HeLa cells with indicated RIF1 expression and treatments. (HeLa cells described in Fig.S3E). HU: 4mM 24hr. *p* values (two-tailed) calculated by Mann-Whitney test.

(J) (K) Western blot analysis of RIF1-L S2265 phosphorylation in HeLa cells with indicated RIF1 expression and treatments. (HeLa cells described in Fig.S3E). HU: 4mM 24hr. ATRi (VE-821): 1µM 24hr; Chk1i (PF-477736): 1µM 24hr.

(L) Potential antibody recognition issue to explain HU-induced reduction of RIF1-L phosphoS^{2265}. Left: sequence of the phospho-peptide used as the antigen to produce our phosphoS^{2265} antibody. Right: hypothesis that serine residues adjacent to S^{2265} could be phosphorylated upon HU, consequently interfering with the phosphoS^{2265} antibody recognizing its epitope.

(M) Western blot analysis of RIF1-L S2265 phosphorylation in total RIF1 population (Input), BRCA1-BRCT-bound RIF1 fraction (mCherry IP), and unbound RIF1 fraction (Flow through). HeLa cells expressing RIF-L or RIF1-L-pp1bs were transfected with mCherry-BRCA1-BRCT plasmid. Whole cell extract, mCherry-IP, and flow through fractions were analysed for RIF1-L-phosphoS2265 and RIF1 (total) immunoblotting.

(N) Quantification of RIF1-L-phosphoS2265 enrichment (calculated as phosphoS2265 WB signal intensity/total RIF1 intensity, normalised to Input samples) in the three fractions as shown in (M). Mean (indicated by number outside end of bars) and standard errors of two independent experiments were plotted.

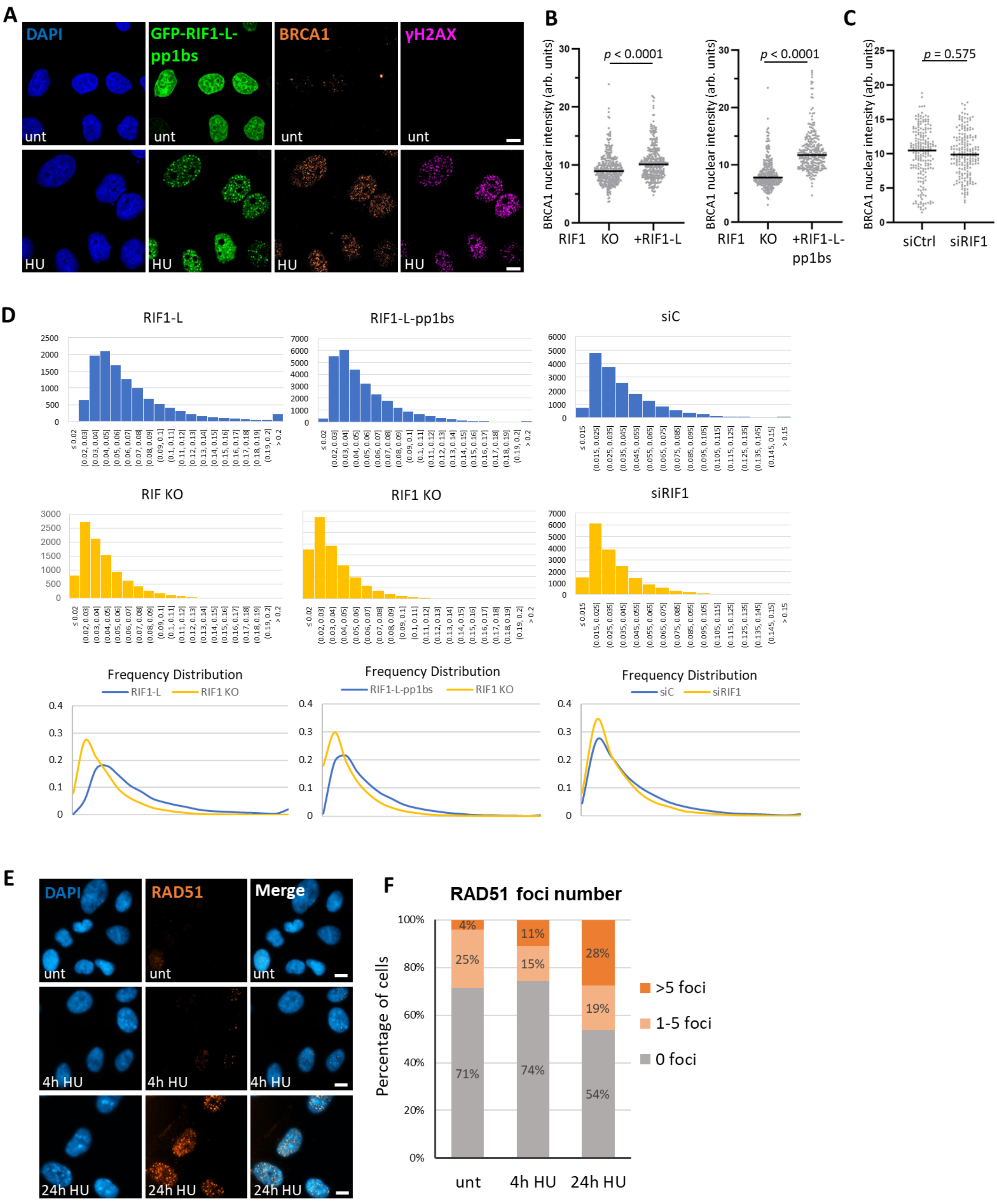

Supplementary Figure 6. RIF1-L interacts with BRCA1 at broken replication forks

(A) Representative images of GFP-RIF1-L-pp1bs, BRCA1, and γH2AX in HeLa cells with indicated treatments. (HeLa cells described in Fig.S3E). BRCA1 and γH2AX signals were generated by immunostaining. unt: not treated with HU; HU: 4mM 24hr. Scalebar: 10µm.

(B) (C) Quantification of BRCA1 nuclear immunofluorescence signal intensity in cells from one of the experiments in Fig.6(C)(D). Black lines represent median values. Three independent experiments were performed, and one representative result is shown. arb.units is arbitrary unit of pixel intensity. *p* values (two-tailed) calculated by Mann-Whitney test. Source data are provided as a Source Data file.

(D) Histograms of individual BRCA1 focus intensity in cells from the experiment as Fig.6(C),(D).

(E) Representative images of RAD51 immunofluorescence signal in HeLa cells with indicated treatments. HU: 4mM, 4 or 24hr. Scalebar: 10µm.

(F) Percentage of cells containing indicated number of RAD51 foci, analysed in cells from the experiment as (D).

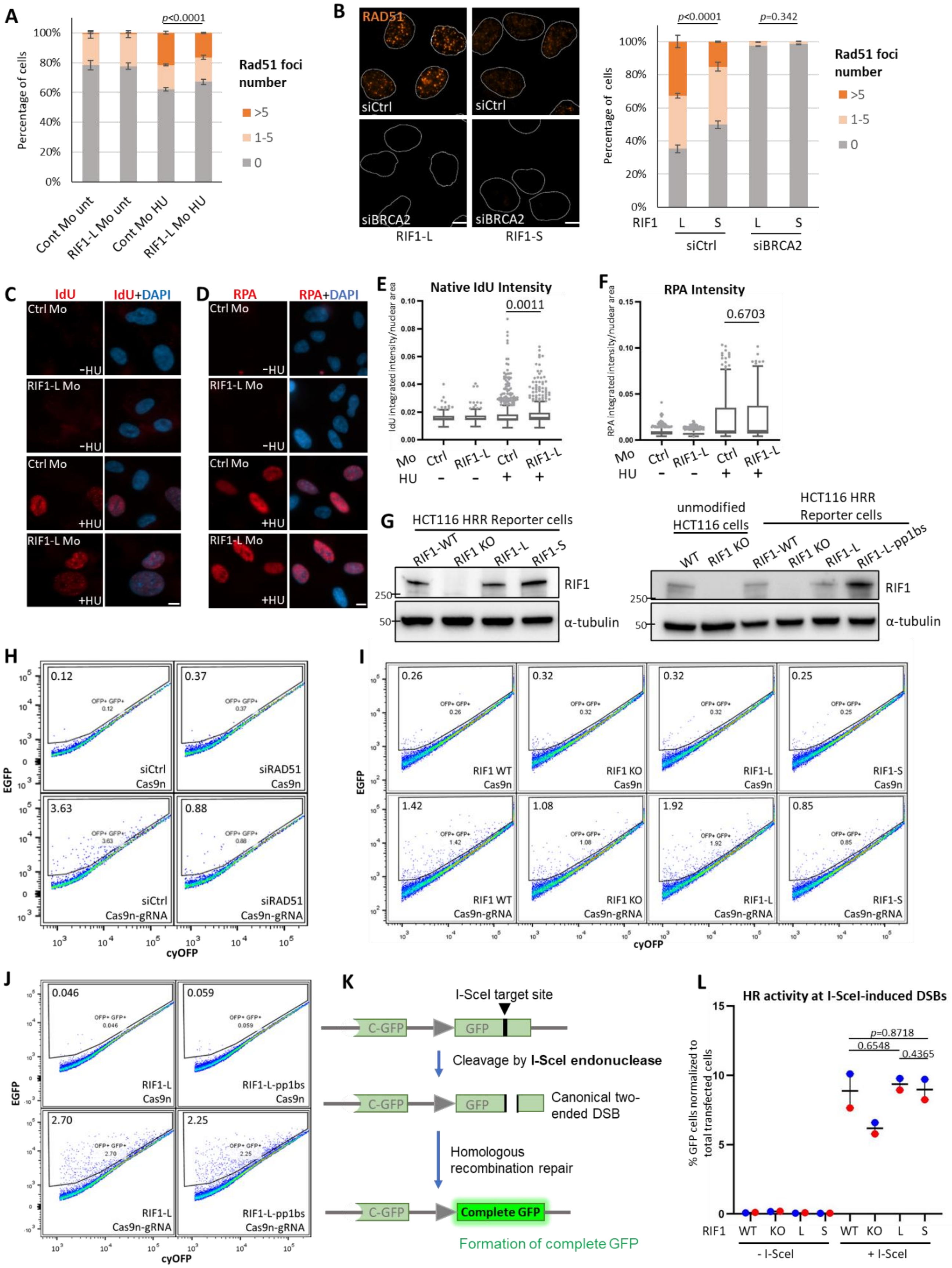

A
Percentage of cells
p<0.0001
Rad51 foci number
>5
1-5
0
Cont Mo unt
RIF1-L Mo unt
Cont Mo HU
RIF1-L Mo HU
B
RAD51
siCtrl
siBRCA2
RIF1-L
RIF1-S
p<0.0001
p=0.342
RIF1
siCtrl
siBRCA2
C
IdU
IdU+DAPI
Ctrl Mo
RIF1-L Mo
-HU
+HU
D
RPA
RPA+DAPI
E
Native IdU Intensity
0.0011
IdU integrated intensity/nuclear area
F
RPA Intensity
0.6703
RPA integrated intensity/nuclear area
Mo
HU
G
HCT116 HRR Reporter cells
RIF1-WT
RIF1 KO
RIF1-L
RIF1-S
RIF1
α-tubulin
unmodified HCT116 cells
HCT116 HRR Reporter cells
WT
RIF1-L-pp1bs
H
EGFP
cyOFP
siCtrl
siRAD51
Cas9n
Cas9n-gRNA
I
RIF1 WT
J
K
I-SceI target site
C-GFP
GFP
Cleavage by I-SceI endonuclease
Canonical two-ended DSB
Homologous recombination repair
Complete GFP
Formation of complete GFP
L
HR activity at I-SceI-induced DSBs
% GFP cells normalized to total transfected cells
p=0.8718
0.6548
0.4365
- I-SceI
+ I-SceI

Supplementary Figure 7. RIF1-L promotes RAD51-dependent repair of broken replication forks

(A) Percentage of nuclei containing indicated number of RAD51 foci in RPE-1 cells with indicated treatments. Mo: abbreviation for Morpholino (as described in Fig.3D-E). unt: not treated with HU; HU: 4mM 24hr. Mean and standard errors of two independent experiments are plotted. *p* value calculated by chi-square test.

(B) Left: Representative images of RAD51 immunofluorescence in HeLa cells that express only RIF1-L or RIF1-S. Cells were treated with either siControl or siBRCA2. All samples were treated with 4mM 24hr HU. Scalebar: 10μm. Right: Percentage of nuclei containing indicated number of RAD51 foci in cells as in left panel. Mean and standard errors of two independent experiments are plotted. *p* values calculated by chi-square test. Source data are provided as a Source Data file.

(C) Representative images of native IdU immunofluorescence in RPE-1 cells with indicated treatments. IdU immunofluorescence was performed in non-denaturing conditions. Mo: abbreviation for Morpholino HU: 4mM 24hr. Scalebar: 10μm.

(D) Representative images of RPA immunofluorescence in RPE-1 cells with indicated treatments. HU: 4mM 24hr. Scalebar: 10μm.

(E) Quantification of native IdU nuclear signal intensity in cells as the experiment in (C). *p* values (two-tailed) calculated by Mann-Whitney test.

(F) Quantification of RPA nuclear signal intensity in cells as the experiment in (D). *p* values (two-tailed) calculated by Mann-Whitney test.

(G) Western blot analysis of RIF1 expression in the HCT116 HR reporter cell lines described in Fig.7F. In these HCT116 HR reporter cells the endogenous RIF1 genes were either deleted (RIF1 KO), or modified by CRISPR to encode specific RIF1 derivatives (RIF1-L, RIF1-S, or RIF1-L-pp1bs) as described.[24]

(H) Flow cytometry analysis using the HCT116 HR reporter cell line described in Fig.7F. The reporter cells were transfected with indicated siRNAs and Cas9n-expressing plasmids (without or with gRNA to target Cas9n at the nicking site). A cyOFP plasmid was co-transfected, enabling successfully transfected cells to be tracked as the cyOFP+ population. The percentage of GFP+ population (indicative of cells that repaired the reporter construct via HR) within the cyOFP+ cells was quantified, and shown as the number at top-left corner in each grid.

(I) Flow cytometry analysis from the experiment described in Fig.7F-G.

(J) Flow cytometry analysis of the HCT116 HR reporter cells (described in Fig.7F) that express either RIF1-L or RIF1-L-pp1bs.

(K) Schematic diagram of reporter construct to assess homologous recombination-mediated repair at I-SceI-induced two-ended DSBs.

(L) Flow cytometry analysis of HR-mediated repair of canonical DSBs assessed by the reporter shown in (H), in cells with indicated RIF1 expression. Means and standard errors of two independent experiments are plotted. *p* values calculated by two-tailed paired Student's t test. Source data are provided as a Source Data file.

**Wavelength**	0.91800
**Resolution range**	56.55 - 1.31 (1.357 - 1.31)
**Space group**	P 1 21 1
**Unit cell**	36.8402 173.716 74.5222 90 89.0457 90
**Total reflections**	566489 (36635)
**Unique reflections**	219241 (20958)
**Multiplicity**	2.6 (1.7)
**Completeness (%)**	98.06 (93.01)
**Mean I/sigma(I)**	9.12 (0.46)
**Wilson B-factor**	18.63
**R-merge**	0.06504 (0.9694)
**R-meas**	0.07965 (1.296)
**R-pim**	0.04537 (0.8519)
**CC1/2**	0.995 (0.326)
**CC***	0.999 (0.702)
**Reflections used in refinement**	218995 (20742)
**Reflections used for R-free**	10833 (991)
**R-work**	0.1409 (0.3301)
**R-free**	0.1786 (0.3613)
**CC(work)**	0.971 (0.610)
**CC(free)**	0.967 (0.573)
**Number of non-hydrogen atoms**	8484
**macromolecules**	7314
**ligands**	407
**solvent**	1005
**Protein residues**	875
**RMS(bonds)**	0.009
**RMS(angles)**	1.01
**Ramachandran favored (%)**	97.87
**Ramachandran allowed (%)**	2.13
**Ramachandran outliers (%)**	0.00
**Rotamer outliers (%)**	0.62
**Clashscore**	1.94
**Average B-factor**	27.03
**macromolecules**	24.86
**ligands**	41.92
**solvent**	40.36

Supplementary Table 1. X-ray diffraction data collection and refinement statistics

Statistics for the highest resolution shell are shown in parentheses.
