# Supplementary material for: The human RIF1-Long isoform interacts with BRCA1 to promote recombinational fork repair under DNA replication stress
Source: Nat Commun. 2025 Jul 1;16:5820. doi: 10.1038/s41467-025-60817-y (PMC12214830; doi:10.1038/s41467-025-60817-y)
Supplement: Supplementary file 2 — Description of Additional Supplementary Files [file 41467_2025_60817_MOESM2_ESM.docx]

**The human RIF1-Long isoform interacts with BRCA1 to promote recombinational fork repair under DNA replication stress**

**Description of Additional Supplementary Files**

File Name: Supplementary Data 1

Description: Sample number n, Mean, and Median values of all box-and-whisker and dot plots in this study. Numbers are listed correspondent to samples from left to right in each plot.

File Name: Supplementary Data 2

Description: Gating Strategy for flow cytometry analyses
