# Supplementary material for: The human RIF1-Long isoform interacts with BRCA1 to promote recombinational fork repair under DNA replication stress
Source: Nat Commun. 2025 Jul 1;16:5820. doi: 10.1038/s41467-025-60817-y (PMC12214830; doi:10.1038/s41467-025-60817-y)
Supplement: Supplementary file 4 — Supplementary Data 2 [file 41467_2025_60817_MOESM4_ESM.pdf]

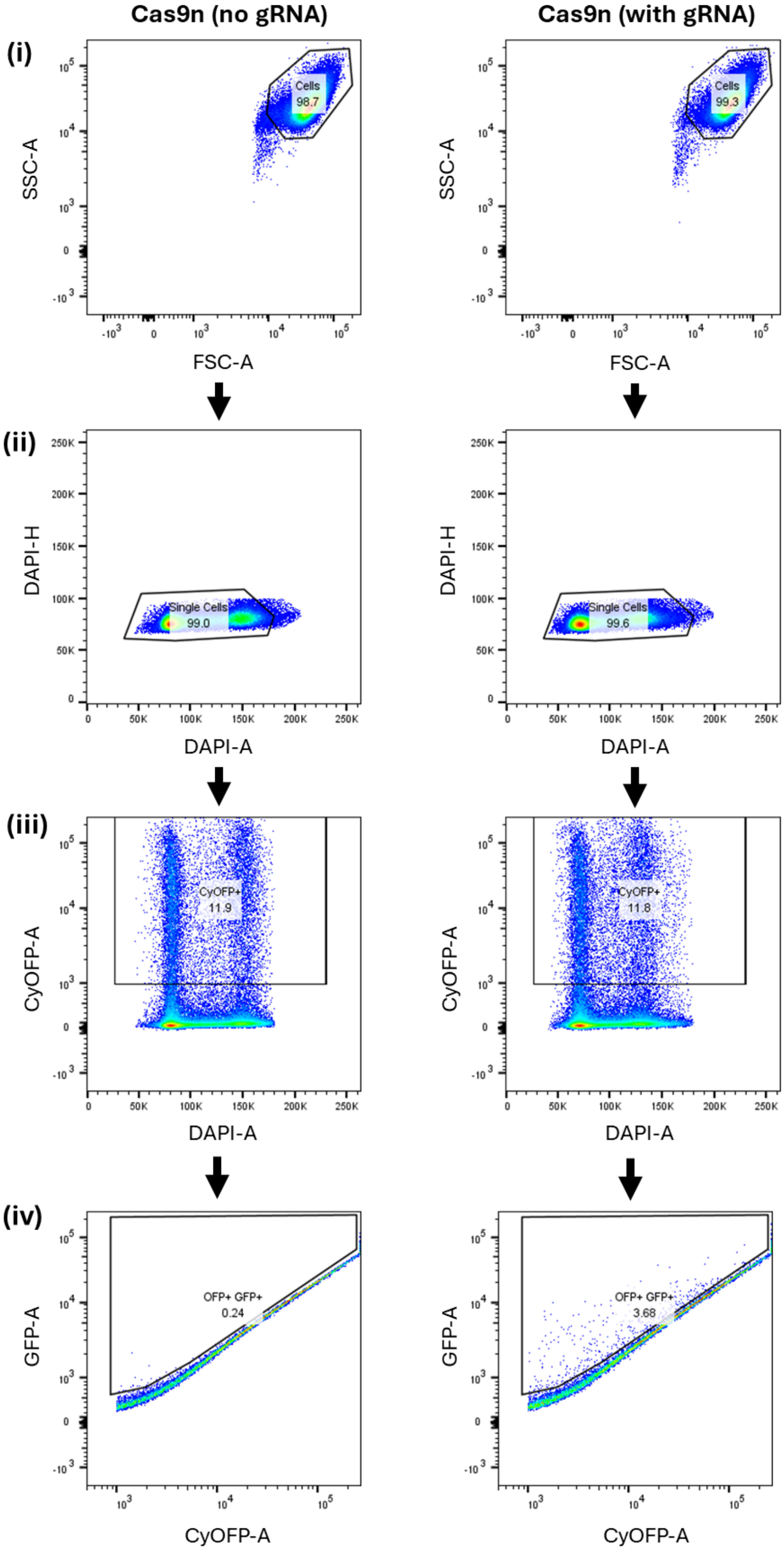

Supplementary Data 2. Gating strategy for flow cytometry analyses

Gating strategy for flow cytometry results presented in Figure 7G and Supplementary Figures 7H-L. **(i)** FSC-A/SSC-A was used to gate cells. **(ii)** DAPI-A/DAPI-H was used to gate single cells. **(iii)** Cells with CyOFP value > 10^3 were defined CyOFP+ population. **(iv)** Within the CyOFP+ population, OFP+ GFP+ gate was defined as having less than 0.5% of GFP+ cells in the Cas9n (no gRNA) control sample.
