# Supplementary material for: The human RIF1-Long isoform interacts with BRCA1 to promote recombinational fork repair under DNA replication stress
Source: Nat Commun. 2025 Jul 1;16:5820. doi: 10.1038/s41467-025-60817-y (PMC12214830; doi:10.1038/s41467-025-60817-y)
Supplement: Supplementary file 5 — Reporting Summary [file 41467_2025_60817_MOESM5_ESM.pdf]

nature portfolio

Corresponding author(s): Anne D. Donaldson

Last updated by author(s): May 18, 2025

Reporting Summary

Nature Portfolio wishes to improve the reproducibility of the work that we publish. This form provides structure for consistency and transparency in reporting. For further information on Nature Portfolio policies, see our Editorial Policies and the Editorial Policy Checklist.

Statistics

For all statistical analyses, confirm that the following items are present in the figure legend, table legend, main text, or Methods section.

n/a	Confirmed	
☐	☒	The exact sample size (*n*) for each experimental group/condition, given as a discrete number and unit of measurement
☐	☒	A statement on whether measurements were taken from distinct samples or whether the same sample was measured repeatedly
☐	☒	The statistical test(s) used AND whether they are one- or two-sided<br>*Only common tests should be described solely by name; describe more complex techniques in the Methods section.*
☒	☐	A description of all covariates tested
☐	☒	A description of any assumptions or corrections, such as tests of normality and adjustment for multiple comparisons
☐	☒	A full description of the statistical parameters including central tendency (e.g. means) or other basic estimates (e.g. regression coefficient) AND variation (e.g. standard deviation) or associated estimates of uncertainty (e.g. confidence intervals)
☐	☒	For null hypothesis testing, the test statistic (e.g. *F*, *t*, *r*) with confidence intervals, effect sizes, degrees of freedom and *P* value noted<br>*Give P values as exact values whenever suitable.*
☒	☐	For Bayesian analysis, information on the choice of priors and Markov chain Monte Carlo settings
☒	☐	For hierarchical and complex designs, identification of the appropriate level for tests and full reporting of outcomes
☒	☐	Estimates of effect sizes (e.g. Cohen's *d*, Pearson's *r*), indicating how they were calculated

Our web collection on statistics for biologists contains articles on many of the points above.

Software and code

Policy information about availability of computer code

Data collection	*Zeiss Zen (Blue edition) was used to acquire images with Zeiss Axio Microscope. Zeiss Zen (Black edition) was used to acquire images with Zeiss LSM880 Microscope. BDFACSDiva was used to acquire flow cytometry data with BD Fortessa flow cytometer. CLARIOstar was used to acquire fluorescence polarisation data with CLARIOstar multimode microplate reader. Diamond Synchrotron Lightsource was employed to collect crystallography data.*
Data analysis	Image Lab was used to process Western blotting and DNA gel electrophoresis images. CellProfiler v3.1.9 and v4.2.6 were used to process fluorescence microscopy images acquired from 1) RIF1-BRCA1 proximity ligation assays - to count PLA foci number, 2) immunofluorescence and fluorescence protein experiments - to count protein foci number, measure protein fluorescence intensity, and perform co-localisation analyses, 3) micronuclei frequency analysis - to count micronuclei number. Custom-developed CellProfiler pipelines are available at .the publicly accessible repository Zenodo [https://zenodo.org/records/15407921]. Flowjo X was used to analyze flow cytometry data. Prism GraphPad (v9) was used to generate box and whisker plots, dot plots, and bar charts for all relevant experiments. Prism GraphPad (v10) was used to generate fluorescence polarisation binding curves. Microsoft Excel was used to generate line charts for colony survival assays and histograms for protein foci intensity distribution. PHASER and PHENIX software packages were used to determine crystal structure. PyMOL v2.2.2 was used to produce structural figures. Alphafold2 v2.3.2 was used to predict RIF1-L protein structure.<br>For statistical analysis, Kruskal-Wallis test with Dunn's multiple comparisons and Mann-Whitney tests were performed using Prism GraphPad (v9). Student's t tests were performed using Excel T.TEST function. Chi-square tests were performed using the online Chi-square test calculator at https://www.socscistatistics.com/tests/chisquare2/default2.aspx

For manuscripts utilizing custom algorithms or software that are central to the research but not yet described in published literature, software must be made available to editors and reviewers. We strongly encourage code deposition in a community repository (e.g. GitHub). See the Nature Portfolio guidelines for submitting code & software for further information.

Data

Policy information about availability of data

All manuscripts must include a data availability statement. This statement should provide the following information, where applicable:

- Accession codes, unique identifiers, or web links for publicly available datasets
- A description of any restrictions on data availability
- For clinical datasets or third party data, please ensure that the statement adheres to our policy

Structure factors and refined atomic coordinates have been deposited in the Protein Data Bank with accession code PDB:8RS8, available at: https://www.rcsb.org/structure/unreleased/8RS8. Raw data underlying box-and-whisker plots, dot plots, bar charts and line graphs is deposited to figshare at https://doi.org/10.6084/m9.figshare.27918258, and available in the Source data file. Uncropped images of DNA gels, protein gels, and Western blots are provided in the Source data file. No restriction applies to data generated in this study.

Research involving human participants, their data, or biological material

Policy information about studies with human participants or human data. See also policy information about sex, gender (identity/presentation), and sexual orientation and race, ethnicity and racism.

Reporting on sex and gender	*No human participant were involved in this study.*
Reporting on race, ethnicity, or other socially relevant groupings	*N/A*
Population characteristics	*N/A*
Recruitment	*N/A*
Ethics oversight	*N/A*

Note that full information on the approval of the study protocol must also be provided in the manuscript.

Field-specific reporting

Please select the one below that is the best fit for your research. If you are not sure, read the appropriate sections before making your selection.

☒ Life sciences ☐ Behavioural & social sciences ☐ Ecological, evolutionary & environmental sciences

For a reference copy of the document with all sections, see nature.com/documents/nr-reporting-summary-flat.pdf

Life sciences study design

All studies must disclose on these points even when the disclosure is negative.

Sample size	No specific method was used to determine sample size. For all relevant experiments, sample size was the number of identified nuclei in 15-25 images acquired with microscope. Sample size is at least 100 to be sufficient for statistical analysis, as detailed in Supplementary Data 1.
Data exclusions	No data were excluded from the analyses.
Replication	*Experiments were performed for at least twice independently and generated reproducible results.*
Randomization	*Randomization was not relevant to this study.*
Blinding	*The investigator was not blinded during data collection and analysis. Blinding was not necessary for this study as data analysis methods are automated and unbiased.*

Behavioural & social sciences study design

All studies must disclose on these points even when the disclosure is negative.

Study description	*Briefly describe the study type including whether data are quantitative, qualitative, or mixed-methods (e.g. qualitative cross-sectional, quantitative experimental, mixed-methods case study).*
Research sample	*State the research sample (e.g. Harvard university undergraduates, villagers in rural India) and provide relevant demographic*

Research sample	*information (e.g. age, sex) and indicate whether the sample is representative. Provide a rationale for the study sample chosen. For studies involving existing datasets, please describe the dataset and source.*
Sampling strategy	*Describe the sampling procedure (e.g. random, snowball, stratified, convenience). Describe the statistical methods that were used to predetermine sample size OR if no sample-size calculation was performed, describe how sample sizes were chosen and provide a rationale for why these sample sizes are sufficient. For qualitative data, please indicate whether data saturation was considered, and what criteria were used to decide that no further sampling was needed.*
Data collection	*Provide details about the data collection procedure, including the instruments or devices used to record the data (e.g. pen and paper, computer, eye tracker, video or audio equipment) whether anyone was present besides the participant(s) and the researcher, and whether the researcher was blind to experimental condition and/or the study hypothesis during data collection.*
Timing	*Indicate the start and stop dates of data collection. If there is a gap between collection periods, state the dates for each sample cohort.*
Data exclusions	*If no data were excluded from the analyses, state so OR if data were excluded, provide the exact number of exclusions and the rationale behind them, indicating whether exclusion criteria were pre-established.*
Non-participation	*State how many participants dropped out/declined participation and the reason(s) given OR provide response rate OR state that no participants dropped out/declined participation.*
Randomization	*If participants were not allocated into experimental groups, state so OR describe how participants were allocated to groups, and if allocation was not random, describe how covariates were controlled.*

Ecological, evolutionary & environmental sciences study design

All studies must disclose on these points even when the disclosure is negative.

Study description	*Briefly describe the study. For quantitative data include treatment factors and interactions, design structure (e.g. factorial, nested, hierarchical), nature and number of experimental units and replicates.*
Research sample	*Describe the research sample (e.g. a group of tagged Passer domesticus, all Stenocereus thurberi within Organ Pipe Cactus National Monument), and provide a rationale for the sample choice. When relevant, describe the organism taxa, source, sex, age range and any manipulations. State what population the sample is meant to represent when applicable. For studies involving existing datasets, describe the data and its source.*
Sampling strategy	*Note the sampling procedure. Describe the statistical methods that were used to predetermine sample size OR if no sample-size calculation was performed, describe how sample sizes were chosen and provide a rationale for why these sample sizes are sufficient.*
Data collection	*Describe the data collection procedure, including who recorded the data and how.*
Timing and spatial scale	*Indicate the start and stop dates of data collection, noting the frequency and periodicity of sampling and providing a rationale for these choices. If there is a gap between collection periods, state the dates for each sample cohort. Specify the spatial scale from which the data are taken*
Data exclusions	*If no data were excluded from the analyses, state so OR if data were excluded, describe the exclusions and the rationale behind them, indicating whether exclusion criteria were pre-established.*
Reproducibility	*Describe the measures taken to verify the reproducibility of experimental findings. For each experiment, note whether any attempts to repeat the experiment failed OR state that all attempts to repeat the experiment were successful.*
Randomization	*Describe how samples/organisms/participants were allocated into groups. If allocation was not random, describe how covariates were controlled. If this is not relevant to your study, explain why.*
Blinding	*Describe the extent of blinding used during data acquisition and analysis. If blinding was not possible, describe why OR explain why blinding was not relevant to your study.*

Did the study involve field work? ☐ Yes ☐ No

Field work, collection and transport

Field conditions	*Describe the study conditions for field work, providing relevant parameters (e.g. temperature, rainfall).*
Location	*State the location of the sampling or experiment, providing relevant parameters (e.g. latitude and longitude, elevation, water depth).*
Access & import/export	*Describe the efforts you have made to access habitats and to collect and import/export your samples in a responsible manner and in compliance with local, national and international laws, noting any permits that were obtained (give the name of the issuing authority, the date of issue, and any identifying information).*

Disturbance | Describe any disturbance caused by the study and how it was minimized.

Reporting for specific materials, systems and methods

We require information from authors about some types of materials, experimental systems and methods used in many studies. Here, indicate whether each material, system or method listed is relevant to your study. If you are not sure if a list item applies to your research, read the appropriate section before selecting a response.

Materials & experimental systems

n/a	Involved in the study	
☐	☒	Antibodies
☐	☒	Eukaryotic cell lines
☒	☐	Palaeontology and archaeology
☒	☐	Animals and other organisms
☒	☐	Clinical data
☒	☐	Dual use research of concern
☒	☐	Plants

Methods

n/a	Involved in the study	
☒	☐	ChIP-seq
☐	☒	Flow cytometry
☒	☐	MRI-based neuroimaging

Antibodies

Antibodies used

anti-RIF1 (Bethyl Laboratories, A300-568A); anti-RIF1-L-Phospho-S2265 (amsbio, custom); anti-BRCA1 (D9) (Santa Cruz Biotechnology, sc-6954); anti-BARD1 (Bethyl Laboratories, A300-263A); anti-53BP1 (Novus Biologicals, NB100-94); anti-γH2A.X (20E3) (Cell Signaling Technology, 9718); anti-RPA32/RPA2 [9H8] (Abcam, ab2175); anti-RAD51 (H92) (Santa Cruz Biotechnology, sc-8349); anti-BrdU (B44) (BD Biosciences, 347580); anti-PP1α (G4) (Santa Cruz Biotechnology, sc-271762); anti-Tubulin (YOL1/34) (Santa Cruz Biotechnology, sc-53030); anti-FLAG (M2) (Sigma-Aldrich, F1804); anti-GFP (Chromotek, 3h9); anti-RFP (Chromotek, 5f8).

Validation

anti-RIF1 (Bethyl Laboratories, A300-568A) validated for Western blot in Supplementary Figures 1F, 3E, 7G; validated for proximity ligation assays in Supplementary Figure 3A of this study.
anti-RIF1-L-Phospho-S2265 (amsbio, custom) validated for phospho-specificity in Supplementary Figure 5D; validated for Western blot in Supplementary Figures 5B, 5C of this study.
anti-BRCA1 (Santa Cruz Biotechnology, sc-6954) validated for Western blot and Immunolfuorescence at product website https://www.scbt.com/p/brca1-antibody-d-9; validated for proximity ligation assays in Supplementary Figure 3A of this study.
anti-BARD1 (Bethyl Laboratories, A300-263A) validated for Western blot at product website https://www.thermofisher.com/antibody/product/BARD1-Antibody-Polyclonal/A300-263A-T.
anti-53BP1 (Novus Biologicals, NB100-94) validated for Western blot in Figure 4A of this study and at product website https://www.novusbio.com/products/53bp1-antibody_nb100-904.
anti-γH2A.X (20E3) (Cell Signaling Technology, 9718) validated for Immunofluorescence at product website https://www.cellsignal.com/products/primary-antibodies/phospho-histone-h2a-x-ser139-20e3-rabbit-mab/9718
anti-RPA32/RPA2 [9H8] (Abcam, ab2175) validated for Immunofluorescence in Garzón et al., Human RIF1-Protein Phosphatase 1 Prevents Degradation and Breakage of Nascent DNA on Replication Stalling. Cell Rep 27, (2019). doi: 10.1016/j.celrep.2019.05.002
anti-RAD51 (H92) (Santa Cruz Biotechnology, sc-8349) validated for Immunofluorescence in Garzón et al., Human RIF1-Protein Phosphatase 1 Prevents Degradation and Breakage of Nascent DNA on Replication Stalling. Cell Rep 27, (2019). DOI: 10.1016/j.celrep.2019.05.002
anti-BrdU (B44) (BD Biosciences, 347580) validated for Immunofluorescence in Vujanovic et al., Replication Fork Slowing and Reversal upon DNA Damage Require PCNA Polyubiquitination and ZRANB3 DNA Translocase Activity. Mol Cell. 2017 Sep 7;67(5):882-890.e5. doi: 10.1016/j.molcel.2017.08.010.
anti-PP1α (G4) (Santa Cruz Biotechnology, sc-271762) validated for Western blot in Supplementary Figure 5E of this study and at product website https://www.scbt.com/p/pp1alpha-antibody-g-4.
anti-Tubulin (YOL1/34) (Santa Cruz Biotechnology, sc-53030) validated for Western blot at product website https://www.scbt.com/p/alpha-tubulin-antibody-yol1-34.
anti-FLAG (M2) (Sigma-Aldrich, F1804) validated for Western blot and IP at product website https://www.sigmaaldrich.com/GB/en/product/sigma/f1804?msockid=3c9d36e2b6456c3a0d6025d4b7d26d98.
anti-GFP (Chromotek, 3h9) validated for Western blot at product website https://www.ptglab.com/products/GFP-antibody-3H9.htm.
anti-RFP (Chromotek, 5f8) validated for Western blot at product website https://www.ptglab.com/products/RFP-antibody-5F8.htm.

Eukaryotic cell lines

Policy information about cell lines and Sex and Gender in Research

Cell line source(s)

HEK293 Flp-In T-REx 293 GFP-RIF1-S (Hiraga et al., 2017)
HEK293 Flp-In T-REx 293 GFP-RIF1-S-pp1bs (Hiraga et al., 2017)
HEK293 Flp-In T-REx 293 GFP-RIF1-L (Watts et al., 2020)
HEK293 Flp-In T-REx 293 GFP-RIF1-L-pp1bs (Watts et al., 2020)
HEK293 Flp-In T-REx 293 GFP-RIF1-L-APKF (Watts et al., 2020)
HEK293 Flp-In T-REx 293 GFP-RIF1-L-pp1bs-APKF (Watts et al., 2020)
HCT116 (ATCC CCL-247)

	HCT116 RIF1 KO (Watts et al., 2020)<br>HCT116 OsTIR1 mAC-RIF1 (Watts et al., 2020)<br>HCT116 OsTIR1 mAC-RIF1-S (Watts et al., 2020)<br>HCT116 OsTIR1 mAC-RIF1-L (Watts et al., 2020)<br>HCT116 HR Reporter RIF1-WT (This study)<br>HCT116 HR Reporter RIF1 KO (This study)<br>HCT116 HR Reporter RIF1-L (This study)<br>HCT116 HR Reporter RIF1-S (This study)<br>HCT116 HR Reporter RIF1-L-pp1bs (This study)<br>hTERT-RPE-1 (ATCC CRL-4000)<br>HeLa (ECACC 93021013)<br>HeLa RIF1 KO (Isobe et al., 2021)<br>HeLa RIF1 KO + GFP-RIF1-L (Isobe et al., 2021)<br>HeLa RIF1 KO + GFP-RIF1-L-pp1bs (Isobe et al., 2021)<br>HeLa RIF1 KO + GFP-RIF1-S (This study)<br>HeLa RIF1 KO + GFP-RIF1-L-APKF (This study)<br>HeLa RIF1 KO + GFP-RIF1-L-pp1bs-APKF (This study)<br>U2OS (ATCC HTB-96)
Authentication	Cell lines obtained from ATCC and ECACC were previously authenticated by STR analyses. Genome modifications made in this study were validated by PCR and/or Sanger. RIF1 KO in HCT 116 was confirmed by Illumina sequencing.
Mycoplasma contamination	All cell lines tested negative for mycoplasma.
Commonly misidentified lines<br>(See ICLAC register)	No commonly misidentified lines were used in this study.

Palaeontology and Archaeology

Specimen provenance	Provide provenance information for specimens and describe permits that were obtained for the work (including the name of the issuing authority, the date of issue, and any identifying information). Permits should encompass collection and, where applicable, export.
Specimen deposition	Indicate where the specimens have been deposited to permit free access by other researchers.
Dating methods	If new dates are provided, describe how they were obtained (e.g. collection, storage, sample pretreatment and measurement), where they were obtained (i.e. lab name), the calibration program and the protocol for quality assurance OR state that no new dates are provided.

☐ Tick this box to confirm that the raw and calibrated dates are available in the paper or in Supplementary Information.

Ethics oversight	Identify the organization(s) that approved or provided guidance on the study protocol, OR state that no ethical approval or guidance was required and explain why not.

Note that full information on the approval of the study protocol must also be provided in the manuscript.

Animals and other research organisms

Policy information about studies involving animals; ARRIVE guidelines recommended for reporting animal research, and Sex and Gender in Research

Laboratory animals	For laboratory animals, report species, strain and age OR state that the study did not involve laboratory animals.
Wild animals	Provide details on animals observed in or captured in the field; report species and age where possible. Describe how animals were caught and transported and what happened to captive animals after the study (if killed, explain why and describe method; if released, say where and when) OR state that the study did not involve wild animals.
Reporting on sex	Indicate if findings apply to only one sex; describe whether sex was considered in study design, methods used for assigning sex. Provide data disaggregated for sex where this information has been collected in the source data as appropriate; provide overall numbers in this Reporting Summary. Please state if this information has not been collected. Report sex-based analyses where performed, justify reasons for lack of sex-based analysis.
Field-collected samples	For laboratory work with field-collected samples, describe all relevant parameters such as housing, maintenance, temperature, photoperiod and end-of-experiment protocol OR state that the study did not involve samples collected from the field.
Ethics oversight	Identify the organization(s) that approved or provided guidance on the study protocol, OR state that no ethical approval or guidance was required and explain why not.

Note that full information on the approval of the study protocol must also be provided in the manuscript.

Clinical data

Policy information about clinical studies

All manuscripts should comply with the ICMJE guidelines for publication of clinical research and a completed CONSORT checklist must be included with all submissions.

Clinical trial registration	*Provide the trial registration number from ClinicalTrials.gov or an equivalent agency.*
Study protocol	*Note where the full trial protocol can be accessed OR if not available, explain why.*
Data collection	*Describe the settings and locales of data collection, noting the time periods of recruitment and data collection.*
Outcomes	*Describe how you pre-defined primary and secondary outcome measures and how you assessed these measures.*

Dual use research of concern

Policy information about dual use research of concern

Hazards

Could the accidental, deliberate or reckless misuse of agents or technologies generated in the work, or the application of information presented in the manuscript, pose a threat to:

No	Yes	
☐	☐	Public health
☐	☐	National security
☐	☐	Crops and/or livestock
☐	☐	Ecosystems
☐	☐	Any other significant area

Experiments of concern

Does the work involve any of these experiments of concern:

No	Yes	
☐	☐	Demonstrate how to render a vaccine ineffective
☐	☐	Confer resistance to therapeutically useful antibiotics or antiviral agents
☐	☐	Enhance the virulence of a pathogen or render a nonpathogen virulent
☐	☐	Increase transmissibility of a pathogen
☐	☐	Alter the host range of a pathogen
☐	☐	Enable evasion of diagnostic/detection modalities
☐	☐	Enable the weaponization of a biological agent or toxin
☐	☐	Any other potentially harmful combination of experiments and agents

Plants

Seed stocks	*Report on the source of all seed stocks or other plant material used. If applicable, state the seed stock centre and catalogue number. If plant specimens were collected from the field, describe the collection location, date and sampling procedures.*
Novel plant genotypes	*Describe the methods by which all novel plant genotypes were produced. This includes those generated by transgenic approaches, gene editing, chemical/radiation-based mutagenesis and hybridization. For transgenic lines, describe the transformation method, the number of independent lines analyzed and the generation upon which experiments were performed. For gene-edited lines, describe the editor used, the endogenous sequence targeted for editing, the targeting guide RNA sequence (if applicable) and how the editor was applied.*
Authentication	*Describe any authentication procedures for each seed stock used or novel genotype generated. Describe any experiments used to assess the effect of a mutation and, where applicable, how potential secondary effects (e.g. second site T-DNA insertions, mosiacism, off-target gene editing) were examined.*

ChIP-seq

Data deposition

☐ Confirm that both raw and final processed data have been deposited in a public database such as GEO.

☐ Confirm that you have deposited or provided access to graph files (e.g. BED files) for the called peaks.

Data access links<br>*May remain private before publication.*	*For "Initial submission" or "Revised version" documents, provide reviewer access links. For your "Final submission" document, provide a link to the deposited data.*
Files in database submission	*Provide a list of all files available in the database submission.*
Genome browser session<br>(e.g. UCSC)	*Provide a link to an anonymized genome browser session for "Initial submission" and "Revised version" documents only, to enable peer review. Write "no longer applicable" for "Final submission" documents.*

Methodology

Replicates	*Describe the experimental replicates, specifying number, type and replicate agreement.*
Sequencing depth	*Describe the sequencing depth for each experiment, providing the total number of reads, uniquely mapped reads, length of reads and whether they were paired- or single-end.*
Antibodies	*Describe the antibodies used for the ChIP-seq experiments; as applicable, provide supplier name, catalog number, clone name, and lot number.*
Peak calling parameters	*Specify the command line program and parameters used for read mapping and peak calling, including the ChIP, control and index files used.*
Data quality	*Describe the methods used to ensure data quality in full detail, including how many peaks are at FDR 5% and above 5-fold enrichment.*
Software	*Describe the software used to collect and analyze the ChIP-seq data. For custom code that has been deposited into a community repository, provide accession details.*

Flow Cytometry

Plots

Confirm that:

☒ The axis labels state the marker and fluorochrome used (e.g. CD4-FITC).

☒ The axis scales are clearly visible. Include numbers along axes only for bottom left plot of group (a 'group' is an analysis of identical markers).

☒ All plots are contour plots with outliers or pseudocolor plots.

☒ A numerical value for number of cells or percentage (with statistics) is provided.

Methodology

Sample preparation	*Cultured human HCT116 cells were treated as desired, fixed with 4%w/v formaldehyde, and stained with DAPI staining to determine DNA content.*
Instrument	*BD Fortessa Cell Analyzer 649225B8*
Software	*BDFACSDiva was used for data acquisition, Flowjo X was used for data analysis.*
Cell population abundance	*100000 singlet cells were acquired for each sample.*
Gating strategy	FSC-A/SSC-A gating was used to identify cells from debris. DAPI-A/DAPI-H was used to identify singlets. cyOFP +/- was used to identify transfected cells. CyOFP value >10^3 is defined as cyOFP +. Within the cyOFP + population, GFP + gating was defined as to have less than 0.5% GFP + cells in control (Cas9n with no gRNA) samples.

☒ Tick this box to confirm that a figure exemplifying the gating strategy is provided in the Supplementary Information.

Magnetic resonance imaging

Experimental design

Design type	*Indicate task or resting state; event-related or block design.*

Design specifications	*Specify the number of blocks, trials or experimental units per session and/or subject, and specify the length of each trial or block (if trials are blocked) and interval between trials.*
Behavioral performance measures	*State number and/or type of variables recorded (e.g. correct button press, response time) and what statistics were used to establish that the subjects were performing the task as expected (e.g. mean, range, and/or standard deviation across subjects).*

Acquisition

Imaging type(s)	*Specify: functional, structural, diffusion, perfusion.*
Field strength	*Specify in Tesla*
Sequence & imaging parameters	*Specify the pulse sequence type (gradient echo, spin echo, etc.), imaging type (EPI, spiral, etc.), field of view, matrix size, slice thickness, orientation and TE/TR/flip angle.*
Area of acquisition	*State whether a whole brain scan was used OR define the area of acquisition, describing how the region was determined.*

Diffusion MRI ☐ Used ☐ Not used

Preprocessing

Preprocessing software	*Provide detail on software version and revision number and on specific parameters (model/functions, brain extraction, segmentation, smoothing kernel size, etc.).*
Normalization	*If data were normalized/standardized, describe the approach(es): specify linear or non-linear and define image types used for transformation OR indicate that data were not normalized and explain rationale for lack of normalization.*
Normalization template	*Describe the template used for normalization/transformation, specifying subject space or group standardized space (e.g. original Talairach, MNI305, ICBM152) OR indicate that the data were not normalized.*
Noise and artifact removal	*Describe your procedure(s) for artifact and structured noise removal, specifying motion parameters, tissue signals and physiological signals (heart rate, respiration).*
Volume censoring	*Define your software and/or method and criteria for volume censoring, and state the extent of such censoring.*

Statistical modeling & inference

Model type and settings	*Specify type (mass univariate, multivariate, RSA, predictive, etc.) and describe essential details of the model at the first and second levels (e.g. fixed, random or mixed effects; drift or auto-correlation).*
Effect(s) tested	*Define precise effect in terms of the task or stimulus conditions instead of psychological concepts and indicate whether ANOVA or factorial designs were used.*

Specify type of analysis: ☐ Whole brain ☐ ROI-based ☐ Both

Statistic type for inference<br>(See Eklund et al. 2016)	*Specify voxel-wise or cluster-wise and report all relevant parameters for cluster-wise methods.*
Correction	*Describe the type of correction and how it is obtained for multiple comparisons (e.g. FWE, FDR, permutation or Monte Carlo).*

Models & analysis

n/a	Involved in the study
☐	☐ Functional and/or effective connectivity
☐	☐ Graph analysis
☐	☐ Multivariate modeling or predictive analysis

Functional and/or effective connectivity	*Report the measures of dependence used and the model details (e.g. Pearson correlation, partial correlation, mutual information).*
Graph analysis	*Report the dependent variable and connectivity measure, specifying weighted graph or binarized graph, subject- or group-level, and the global and/or node summaries used (e.g. clustering coefficient, efficiency, etc.).*
Multivariate modeling and predictive analysis	*Specify independent variables, features extraction and dimension reduction, model, training and evaluation metrics.*
